# T-BET and EOMES Accelerate and Enhance Functional Differentiation of Human Natural Killer Cells

**DOI:** 10.3389/fimmu.2021.732511

**Published:** 2021-09-24

**Authors:** Laura Kiekens, Wouter Van Loocke, Sylvie Taveirne, Sigrid Wahlen, Eva Persyn, Els Van Ammel, Zenzi De Vos, Patrick Matthys, Filip Van Nieuwerburgh, Tom Taghon, Pieter Van Vlierberghe, Bart Vandekerckhove, Georges Leclercq

**Affiliations:** ^1^ Laboratory of Experimental Immunology, Department of Diagnostic Sciences, Ghent University, Ghent, Belgium; ^2^ Cancer Research Institute Ghent (CRIG), Ghent, Belgium; ^3^ Department of Biomolecular Medicine, Ghent University, Ghent, Belgium; ^4^ Laboratory of Immunobiology, Rega Institute for Medical Research, Department of Microbiology, Immunology and Transplantation, K.U. Leuven, Leuven, Belgium; ^5^ Laboratory of Pharmaceutical Biotechnology, Department of Pharmaceutics, Ghent University, Ghent, Belgium

**Keywords:** human NK cells, transcription factors, T-BET, EOMES, CD16 expression, antibody-dependent cellular cytotoxicity, NK cell biology, NK cell therapy

## Abstract

T-bet and Eomes are transcription factors that are known to be important in maturation and function of murine natural killer (NK) cells. Reduced T-BET and EOMES expression results in dysfunctional NK cells and failure to control tumor growth. In contrast to mice, the current knowledge on the role of T-BET and EOMES in human NK cells is rudimentary. Here, we ectopically expressed either T-BET or EOMES in human hematopoietic progenitor cells. Combined transcriptome, chromatin accessibility and protein expression analyses revealed that T-BET or EOMES epigenetically represses hematopoietic stem cell quiescence and non-NK lineage differentiation genes, while activating an NK cell-specific transcriptome and thereby drastically accelerating NK cell differentiation. In this model, the effects of T-BET and EOMES are largely overlapping, yet EOMES shows a superior role in early NK cell maturation and induces faster NK receptor and enhanced CD16 expression. T-BET particularly controls transcription of terminal maturation markers and epigenetically controls strong induction of KIR expression. Finally, NK cells generated upon T-BET or EOMES overexpression display improved functionality, including increased IFN-γ production and killing, and especially EOMES overexpression NK cells have enhanced antibody-dependent cellular cytotoxicity. Our findings reveal novel insights on the regulatory role of T-BET and EOMES in human NK cell maturation and function, which is essential to further understand human NK cell biology and to optimize adoptive NK cell therapies.

## Introduction

Natural killer (NK) cells can provide anti-tumor effects by the production of proinflammatory cytokines and by direct target lysis (1–3). NK cells, like other lymphocytes, originate from CD34^+^ hematopoietic stem cells (HSC) in the bone marrow that differentiate through a common lymphoid progenitor stage. In secondary lymphoid tissues, human NK cell development is pursued, whereby the cells sequentially develop into stage 1 (CD34^+^CD45RA^+^CD117^-^CD94^-^) pro-NK cells, followed by stage 2 or pre-NK cells (CD34^+^CD45RA^+^CD117^+^CD94^-^). Stage 1 and stage 2 cells are multipotent as they also have T-cell and dendritic cell developmental potential. Stage 3 cells (CD34^-^CD117^+^CD94^-^CD16^-^) are committed NK cell precursors since they can no longer develop into T-cells or dendritic cells. Stage 4 (CD34^-^CD56^bright^CD94^+^CD16^-^) and stage 5 (CD34^-^CD56^dim^CD94^+^CD16^+^) are mature NK cells (4–7). Differentiation and functional maturation of NK cells is a complex molecular process tightly regulated by transcription factors. Many essential factors have been identified in the transcriptional control of murine NK cell differentiation, thanks to the generation of transcription factor-deficient mice (8). In contrast to mice, the current knowledge on the role of transcription factors in human NK cell differentiation is extremely limited.

Because of their intrinsic anti-tumor effects, NK cells are promising agents for cancer immunotherapy. Although different approaches using NK cells in cancer therapy have already been used, there are still major limitations. It has been shown that NK cell function after allogeneic hematopoietic stem cell transplantation (HSCT) in leukemia patients is impaired (9). The expression level of the transcription factors T-box expressed in T cells (T-BET) and Eomesodermin (EOMES) expression is strongly reduced in NK cells from HSCT recipients and this is associated with less favorable outcome after HSCT as a result of increased nonrelapse mortality (10). Analysis of adoptively transferred mature NK cells in different murine tumor models has shown that NK cells do traffic to tumor sites, but this is accompanied with loss of effector functions, including cytotoxicity and IFN-γ production, and the cells fail to control tumor growth. This dysfunction is accompanied by downregulation of the transcription factors T-BET and EOMES (11).

T-bet and Eomes are two members of the T-box transcription factor family (12). T-bet, encoded by the *Tbx21* gene, is only expressed in hematopoietic cells and is known as a master regulator of T-cell effector functions, including IFN-γ production and cytotoxicity (13). Eomes plays an important role in vertebrate embryogenesis and shares homology with T-bet. Moreover, T-bet and Eomes play a critical role in differentiation, maintenance and function of murine NK cells (14, 15). T-bet-deficient (T-bet^-/-^) mice show reduced numbers of NK cells in liver, spleen and peripheral blood. In contrast, the number of NK cells in the bone marrow is slightly higher in T-bet^-/-^ mice and these NK cells have an immature phenotype (16, 17). Eomes^flox/flox^Vav-Cre^+^ mice show a more substantial decrease of NK cell numbers in spleen and peripheral blood, but not in liver. Eomes-deficient NK cells also show an immature phenotype. Mice lacking both T-bet and Eomes completely fail to develop NK cells in all organs (17). These knockout mouse models show that both T-bet and Eomes are indispensable for NK cell development and terminal NK cell maturation. In parallel to mice, human peripheral blood and spleen NK cells are characterized by a T-BET and EOMES gradient. As NK cells progress from stage 3 to stage 5, they downregulate EOMES and upregulate T-BET, highlighting their reciprocal relationship. This illustrates that the T-BET and EOMES gradient follows the pattern of NK cell maturation, whereby EOMES^low^T-BET^high^ cells are considered as terminal mature NK cells (18, 19).

As low T-BET and EOMES expression levels in NK cells from tumor patients negatively impacts the anti-tumor effects, we here studied the effects of either T-BET or EOMES overexpression in cord blood-derived hematopoietic progenitor cells (HPC) on NK cell differentiation and function. Transcriptome and chromatin accessibility profiling demonstrate that T-BET or EOMES overexpression in human HPC epigenetically regulates activation of an NK cell transcriptome, leading to drastic acceleration of NK cell differentiation. Furthermore, the early arising NK cells have a mature phenotype and are enriched in CD16 expression. In-depth analysis of mature NK cells generated from T-BET- or EOMES-overexpressing HPC shows that terminal maturation of these NK cells is regulated at the epigenome level, wherein T-BET plays a predominant role. Additionally, NK cells generated from T-BET- or EOMES-overexpressing HPC are functional, whereby EOMES overexpression NK cells display enhanced antibody-dependent cellular cytotoxicity (ADCC). Altogether, these findings give new insights in the regulatory role of T-BET and EOMES in human NK cell differentiation and function that can be used to optimize adoptive NK cell therapies.

## Materials and Methods

### Retroviral Overexpression Constructs

Human T-BET and EOMES cDNA (Source BioScience, Nottingham, UK; T-BET cDNA: IRATp970D0558D; EOMES cDNA: IRAKp961A1269Q) were ligated separately into the LZRS-IRES-eGFP retroviral vector (20). The empty LZRS-IRES-eGFP vector was used as control. Retrovirus was generated as previously described (21).

### Isolation of CD34^+^ HPC From Umbilical Cord Blood

Umbilical cord blood (UCB) was obtained from the Cord Blood Bank, Ghent University Hospital, Ghent, Belgium. Mononuclear cells were obtained by Lymphoprep density gradient centrifugation and CD34^+^ HPC were subsequently enriched using Magnetic Activated Cell Sorting (Direct CD34^+^ HSC MicroBead Kit, Miltenyi Biotech, Leiden, The Netherlands) according to the manufacturer’s guidelines. Enriched CD34^+^ HPC were stored in liquid nitrogen until usage.

### *In Vitro* NK Cell Differentiation Co-Culture

#### Culture of EL08.1D2 Cells

*In vitro* NK cell differentiation co-cultures on the murine embryonic liver cell line EL08.1D2 were performed as described by Cichoki and Miller (22) with minor adaptations. EL08.1D2 feeder cells were maintained in 50% Myelocult M5300 medium (Stem Cell Technologies, Grenoble, France), 35% α-MEM, 15% FCS and 10 μM β-mercaptoethanol (Sigma-Aldrich, Saint Louis, MO), supplemented with 100 U/mL penicillin, 100 μg/mL streptomycin, 2 mM glutamine (all from Life Technologies, Carlsbad, CA) on 0.1% gelatin-coated plates at 33°C and 5% CO2. EL08.1D2 cells were inactivated by adding 10 µg/ml mitomycin C (Sigma-Aldrich) to the culture medium for 2-3 hours. Thereafter, cells were thoroughly rinsed before harvesting using trypsin-EDTA. Cells were plated at a density of 5x10^4^ cells per well on a 0.1% gelatin-coated tissue culture-treated 24-well plate at least 24 h before adding HPC or before transfer of differentiated NK cells on day 14 and day 21 of culture.

#### Retroviral Transduction of HPC and NK Cell Differentiation

Isolated UCB-derived CD34^+^ HPC were cultured in complete IMDM containing 10% FCS and supplemented with thrombopoietin (TPO) (20 ng/ml), stem cell factor (SCF) (100 ng/ml) (all from Peprotech, London, UK.) and FMS-like tyrosine kinase 3 ligand (FLT3-L) (100 ng/ml, R&D Systems, Minneapolis, MN) for 48 hours. Subsequently, these cells were harvested, transferred to RetroNectin (Takara Bio, Saint-Germain-en-Laye, France)-coated plates and viral supernatant was added. Additional cytokines were added to keep the concentrations constant after virus addition. The plates were centrifuged at 950 g and 32°C during 90 min. After 48 hours, eGFP^+^lineage^-^(CD3/CD14/CD19/CD56) CD34^+^ HPC were sorted using a BD FACSAria™ Fusion cell sorter (BD Biosciences, San Jose, CA). Sorted HPC were co-cultured with mitomycin-inactivated EL08.1D2 cells for 3, 7, 14 or 21 days in Dulbecco’s modified Eagle medium plus Ham’s F-12 medium (2:1 ratio), supplemented with 100 U/mL penicillin, 100 μg/mL streptomycin, 2 mM glutamine, 10 mM sodium pyruvate (all from Life Technologies), 20% of heat-inactivated human AB serum (Biowest, Nuaillé, France), 24 μM β-mercaptoethanol, 20 μg/mL ascorbic acid and 50 ng/mL sodium selenite (all from Sigma-Aldrich). The following cytokines were added: IL-3 (5 ng/mL, first week only, R&D systems), IL-7 (20 ng/mL), IL-15 (10 ng/mL) (all from Miltenyi Biotec), SCF (20 ng/mL), and Flt3-L (10 ng/mL). Alternatively, to test the necessity of IL-15 in NK cell differentiation upon T-BET and EOMES transduction, IL-15 was not included in the cytokine mix. Culture medium was refreshed on day 7 by addition of the same volume of fresh medium with cytokines. At day 14 the non-adherent cells were harvested and transferred to new mitomycin C-treated EL08.1D2 feeder cells.

### Library Preparation, RNA Sequencing, and qPCR Confirmation

For transcriptome analysis, day 0 eGFP^+^lineage^-^CD34^+^ HPC, day 21 stage 4 (eGFP^+^CD45^+^CD56^+^CD94^+^CD16^-^) and stage 5 NK cells (eGFP^+^CD45^+^CD56^+^CD94^+^CD16^+^) were sorted and RNA was isolated (RNeasy micro kit, Qiagen, Hilden, Germany). The concentration and quality of the extracted RNA was checked using the ‘Quant-it ribogreen RNA assay’ (Life Technologies) and the RNA 6000 nano chip (Agilent Technologies, Santa Clara, CA, U.S.A), respectively. Subsequently, 71 ng and 59 ng of RNA from HPC samples and NK cell samples, respectively, was used to perform an Illumina sequencing library preparation using the QuantSeq 3’ mRNA-Seq Library Prep Kit (Lexogen, Vienna, Austria) according to manufacturer’s protocol. Libraries were quantified by qPCR, according to Illumina’s protocol ‘Sequencing Library qPCR Quantification protocol guide’, version February 2011. A High sensitivity DNA chip (Agilent Technologies) was used to check the library’s size distribution and quality. Sequencing was performed on a high throughput Illumina NextSeq 500 flow cell generating 75 bp single-end reads. Per sample, on average 5.3x10^6^ ± 1.7x10^5^ and 7.32x10^6^ ± 1.78x10^5^ reads of HPC and NK cell samples, respectively, were generated, whereby quality control of these reads was performed with FastQC (23). Reads were then mapped against the Homo sapiens GRCh38.90 reference genome using STAR version 2.42 and gencode version 25 as guide gtf. Gene quantification was performed on the fly by STAR (24). To explore if the samples from different treatment groups clustered together and to detect outlier samples, a Principal Component Analysis (PCA) on vst transformed counts was performed using the R statistical computing software (25). No outliers among the samples were detected. Differential gene expression analysis was performed using DESeq2 (26) with Wald test, whereby T-BET- or EOMES-overexpressing HPC were compared to control-transduced HPC, or stage 4 and stage 5 NK cells generated upon T-BET or EOMES overexpression were compared to control NK cells. Genes with an FDR<0.1 were considered significantly differential.

GSEA was performed using the GSEA software tool v4.1.0 of the Broad Institute (27, 28). The ‘GSEAPreranked’ module was run using standard parameters and 1000 permutations.

To perform qPCR analysis, RNA was converted to cDNA using Superscript RT III (Life Technologies, Carlsbad, CA). PCR reactions were executed using the LightCycler 480 SYBR Green I Master mix (Roche, Diegem, Belgium) on a LightCycler 480 real-time PCR system (Roche) using the primers indicated in **Table 1**.

**Table 1:** qPCR primers for RNA-seq confirmation.

Gene	Sense	Sequence
*ETS1*	Fwd	5’-AGATGGCTGGGAATTCAAAC-3’
	Rev	5’-TTCCTCTTTCCCCATCTCCT-3’
*IKZF2*	Fwd	5’-GCCGTTCAAATGTCCTTTCTG-3’
	Rev	5’-CTTGTAGCTTCGTCCACAGTAG-3’
*IRF8*	Fwd	5’-ATGTGTGACCGGAATGGTGG-3’
	Rev	5’-AGTCCTGGATACATGCTACTGTC-3’
*RUNX2*	Fwd	5’-GTAGCAAGGTTCAACGATCT-3’
	Rev	5’-GTGAAGACGGTTATGGTCAA-3’
*TOX*	Fwd	5’-TATGTGCCAGCCAGCCAGTCCTA-3’
	Rev	5’-TGGTCTGGGAGGGAAGGAGGAGTAA-3’
*IL2RB*	Fwd	5’-AGACCCCTCGAAGTTCTTTTCC-3’
	Rev	5’-CAGGGCTGAAGGACGATGAG-3’

### Library Preparation and Fast-ATAC Sequencing

Chromatin landscape analysis was performed on the same cell populations as used for RNA sequencing. Library preparation was performed as previously described (29, 30). Briefly, 5x10^4^ HPC or day 21 stage 4 and stage 5 NK cells were sorted using a BD FACSAria™ Fusion cell sorter, washed 2 times with cold PBS and pelleted by centrifugation at 500g for 5 minutes at 4°C. After removal of the supernatant, 50 µl of transposase mixture (25 µl 2x TD buffer, 2.5 µl of TDE1 and 22 µl nuclease free water) (all from Illumina, San Diego, CA) containing 0.5 µl of 1% digitonin (Sigma-Aldrich) was added to the cells. Transposition reactions were incubated at 37°C for 30 min with agitation at 250 rpm. Transposed DNA was then purified using the QIAgen MinElute PCR purification kit (Qiagen, Hilden, Germany) according to the manufacturer’s instructions. Thereafter, dsDNA was qualified and quantified with Qubit™ dsDNA HS Assay Kit and Qubit™ Fluometer (both from Thermo Fisher Scientific). Next, transposed fragments were amplified using 2x Kapa HiFi HOTSTART Ready Mix (Roche) and barcoded primers (listed in **Table 2**) as previously described. After amplification, the Fast-ATAC libraries were again purified with the Zymo DNA Clean & Concentrator-5 kit (Zymo Research, Irvine, CA) and visualized on a 1.5% agarose gel to evaluate DNA fragmentation. Libraries underwent agarose gel size selection (150-1000 bp) and quality control before sequencing. All Fast-ATAC libraries were sequenced using paired-end, dual index sequencing on a high throughput Illumina NextSeq 500 flow cell generating 75 bp paired-end reads. Per sample, 1.58x10^7^ ± 4.28x10^5^ and 1.54x10^7^ ± 5.95x10^5^ reads of HPC and NK cell samples, respectively, were generated, whereby quality control of these reads was performed with FastQC (23). Reads were then mapped to the human GRCh38.90 reference genome using STAR version 2.42 and gencode version 25 as guide gtf. Gene quantification was performed on the fly by STAR (24). PCA on vst transformed counts was executed to detect outliers, using the R statistical computing software (25). Among the samples no outliers were detected. Differential expression analysis was performed with DESeq2 (26) with Wald test, whereby HPC upon T-BET or EOMES overexpression were compared to control HPC or stage 4 and stage 5 NK cells from T-BET or EOMES overexpression cultures were compared to control NK cells. Genes with FDR<0.05 were considered significant differential. 

**Table 2:** Barcoded primers for Fast-ATAC sequencing.

Primer No.	Sense	Index	Sequence^a^
8824	Fwd	no index	AATGATACGGCGACCACCGAGAGATCTACACTCGTCGGCAGCGTCAGATGT*G
8825	Rev	TCCCGA	CAAGCAGAAGACGGCATACGAGATTCGGGAGTCTCGTGGGCTCGGAGATG*T
8826	Rev	TCATTC	CAAGCAGAAGACGGCATACGAGATGAATGAGTCTCGTGGGCTCGGAGATG*T
8827	Rev	CTCAGA	CAAGCAGAAGACGGCATACGAGATTCTGAGGTCTCGTGGGCTCGGAGATG*T
8828	Rev	CTATAC	CAAGCAGAAGACGGCATACGAGATGTATAGGTCTCGTGGGCTCGGAGATG*T
8829	Rev	CTAGCT	CAAGCAGAAGACGGCATACGAGATAGCTAGGTCTCGTGGGCTCGGAGATG*T
8830	Rev	CGGAAT	CAAGCAGAAGACGGCATACGAGATATTCCGGTCTCGTGGGCTCGGAGATG*T
8832	Rev	CATTTT	CAAGCAGAAGACGGCATACGAGATAAAATGGTCTCGTGGGCTCGGAGATG*T
10776	Rev	ACTGAT	CAAGCAGAAGACGGCATACGAGATATCAGTGTCTCGTGGGCTCGGAGATG*T
10777	Rev	ATGAGC	CAAGCAGAAGACGGCATACGAGATGCTCATGTCTCGTGGGCTCGGAGATG*T
10778	Rev	ATTCCT	CAAGCAGAAGACGGCATACGAGATAGGAATGTCTCGTGGGCTCGGAGATG*T
10779	Rev	CAAAAG	CAAGCAGAAGACGGCATACGAGATCTTTTGGTCTCGTGGGCTCGGAGATG*T
10781	Rev	GGTAGC	CAAGCAGAAGACGGCATACGAGATGCTACCGTCTCGTGGGCTCGGAGATG*T

^a^*phosphorothioate linkage to prevent endonuclease digestion of the primer.

Motif enrichment analysis was executed with Homer findMotifsGenome.pl with default settings, whereby a bed file of all detected ATAC peaks was used as background. Homer findMotifsGenome.pl with -find option was used to recover individual motif locations (31).

### Flow Cytometry

Cells were examined using flow cytometry (LSRII flow cytometer, BD Biosciences) and data were analyzed with FACSDiva Version 6.1.2 (BD Biosciences) and/or FlowJo_V10 (Ashland, OR, U.S.A) software. Utilized antibodies are listed in **Table 3**. Before staining, cells were blocked with anti-mouse FcγRII/III (unconjugated, clone 2.4G2, kindly provided by Dr J. Unkeless, Mount Sinai School of Medicine, New York, NY) and anti-human FcR blocking reagent (Miltenyi Biotec). Propidium iodide or the LIVE/DEAD Fixable Aqua Dead Cell Stain Kit (Life Technologies) was used to discriminate live and dead cells. FoxP3/Transcription Factor Staining buffer set (eBioscience, Thermo Fisher Scientific, Waltham, MA) was used to perform transcription factor staining.

**Table 3:** Utilized antibodies for flow cytometry.

Antibody	Alternative name	Conjugated fluorochrome	Clone	Supplier
CD117	CD117	Phycoerythrin-Cyanin7 (PECy7)	104D2	eBioscience™; Thermofisher Scientific
CD11a	LFA-1	Phycoerythrin-Cyanin7 (PECy7)	HI111	Biolegend
CD11a	LFA-1	Phycoerythrin (PE)	HI111	BD Pharmingen
CD11a	LFA-1	Allophycocyanin (APC)	HI111	Biolegend
CD14	CD14	Allophycocyanin (APC)	REA599	Miltenyi Biotec
CD158a/h	KIR2DL1/DS1	Phycoerythrin (PE)	REA1010	Miltenyi Biotec
CD158b1/b2	KIR2DL2/DL3/DS2	Phycoerythrin (PE)	GL183	Beckman Coulter
CD158e1/e2	KIR3DL1/DS1	Phycoerythrin (PE)	Z27.3.7	Beckman Coulter
CD158i	KIR2DS4	Phycoerythrin (PE)	FES172	Beckman Coulter
CD159a	NKG2A	Allophycocyanin (APC)	REA110	Miltenyi Biotec
CD16	CD16	Allophycocyanin (APC)	B73.1	Biolegend
CD16	CD16	Phycoerythrin (PE)	B73.1	Biolegend
CD19	CD19	Allophycocyanin (APC)	SJ25C1	eBioscience™; Thermofisher Scientific
CD226	DNAM-1	Alexa Fluor 647	DX11	BD Pharmingen
CD3	CD3	Allophycocyanin (APC)	SK7	Biolegend
CD314	NKG2D	Allophycocyanin (APC)	1D111	Biolegend
CD335	NKp46	Phycoerythrin-Cyanin7 (PECy7)	9E2	Biolegend
CD336	NKp44	Allophycocyanin (APC)	44.189	eBioscience™; Thermofisher Scientific
CD337	NKp30	Phycoerythrin (PE)	P30-15	Biolegend
CD34	CD34	VioBlue (VB)	AC136	Miltenyi Biotec
CD34	CD34	Phycoerythrin (PE)	AC136	Miltenyi Biotec
CD45	CD45	Allophycocyanin (APC)/Fire™750	2D1	Biolegend
CD45RA	CD45RA	Allophycocyanin (APC)	HI100	Biolegend
CD56	NCAM1	Pacific Blue (PB)	5.1H11	Biolegend
CD56	NCAM1	Allophycocyanin (APC)	5.1H11	Biolegend
CD94	KLRD1	Peridinin Chlorophyll Protein- Cyanin5.5 (PerCP-Cy5,5)	DX22	Biolegend
EOMES	EOMES	Phycoerythrin (PE)	WD1928	eBioscience™; Thermofisher Scientific
Granzyme B	Granzyme B	Phycoerythrin (PE)	GB11	eBioscience™; Thermofisher Scientific
IFN-γ	Interferon-γ	eFluor 660	4S.B3	eBioscience™; Thermofisher Scientific
IKZF2	HELIOS	Allophycocyanin (APC)	22F6	Biolegend
Perforin	Perforin	Phycoerythrin (PE)	delta G9	eBioscience™; Thermofisher Scientific
T-BET	T-BET	Phycoerythrin (PE)	4B10	eBioscience™; Thermofisher Scientific

To determine absolute cell numbers of HPC or NK cell subpopulations, cultured cells were harvested and counted with trypan blue in a Bürker counting chamber to determine the total number of viable cells. These counted viable cell numbers were normalized to a starting cell number of 1000 cells on day 0 for each condition. FACS analysis was used to determine the percentages of the different subpopulations and the normalized viable cell numbers were then multiplied by the corresponding percentage of each subpopulation.

### Cytospins

eGFP^+^CD45^+^CD56^+^CD94^+^ NK cells were sorted from day 3 and 7 T-BET or EOMES overexpression cultures and from day 19 control cultures. Cytospins were made using Shandon Cytospin™ 4 (Thermo Fisher Scientific, Carlsbad, CA) according to the manufacturer’s guidelines. For reliable microscopical discrimination of individual cells, less than 500 cells per µl in a volume of 250 µl was added to the sample chamber of the cytoclips. Dry cytoslides were subsequently Wright-Giemsa stained for microscopical evaluation.

### Functional Assays

#### IFN-γ Production

For intracellular IFN-γ detection, 10^5^ cells from day 21 T-BET or EOMES overexpression and control cultures were stimulated with 50 ng/ml phorbol myristate acetate (PMA) and 1 µg/ml ionomycin (both from Sigma-Aldrich) or with 10 ng/ml IL-12 (PeproTech) and 10 ng/ml IL-18 (R&D Systems) with or without 10 ng/ml IL-15 (Miltenyi Biotec) for 24 h. The final 4 h, brefeldin A (BD GolgiPlug, 1/1000, BD Biosciences) was added. Intracellular IFN-γ staining was performed using Cytofix/Cytoperm Kit (BD Biosciences) and analyzed on gated NK cells by flow cytometry.

#### Cytotoxicity Assays

For cell specific killing K562, Daudi, RL, Raji (all from ATCC, Manassas, VA) and Nalm-6 (DSMZ, Braunschweig, Germany) target cells were challenged with sorted (eGFP^+^CD45^+^CD56^+^CD94^+^) NK cells of day 21 control, T-BET and EOMES overexpression cultures in ^51^Chromium release assays as previously described (32). For ADCC assays, Raji cells were used as targets and added to the effector cells at a ratio of 1:1 with either 0 or 10 µg/ml Rituximab (anti-CD20 antibody; Hoffmann-La Roche, Basel, Switzerland; kindly provided by the pharmacy of Ghent University Hospital, Ghent, Belgium). After 4 hours, specific lysis was calculated as described (32).

### Statistical Analysis

Statistical analysis was performed with GraphPad Prism 9.0.0 software (San Diego, CA) using Wilcoxon signed rank test or t-test. P-values of p<0.05 were considered as statistically significant.

## Results

### Epigenetic Regulation by T-BET or EOMES Overexpression in HPC Results in an NK Cell Transcriptome

**Figure 1:**
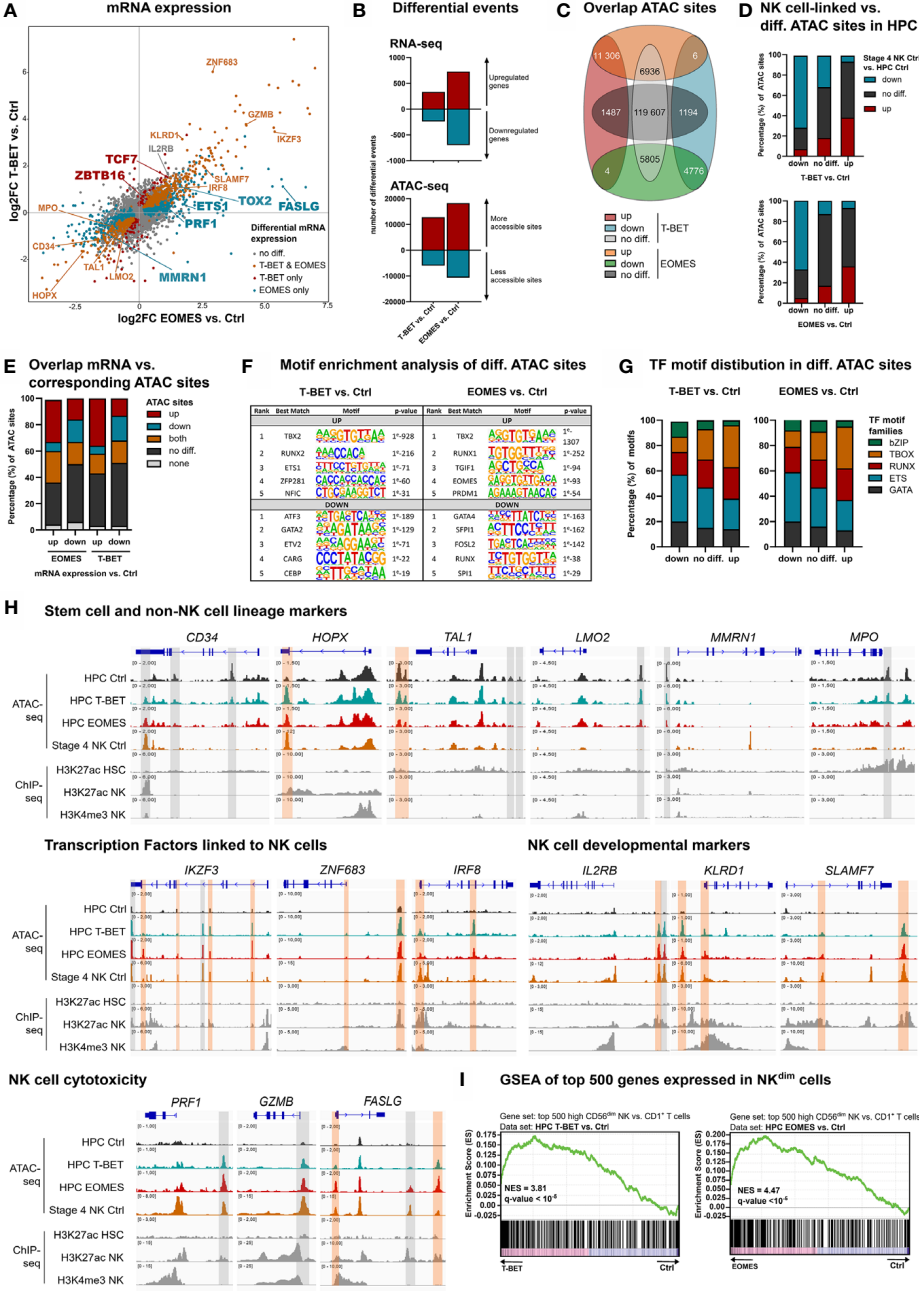
Epigenetic regulation by T-BET or EOMES overexpression in human HPC results in an NK cell transcriptome. Human eGFP^+^CD34^+^ HPC transduced with control, T-BET or EOMES constructs were sorted at day 0 (after 48 h of transduction) and RNA-seq (n=5) and Fast-ATAC-seq (n=3) was performed. **(A)** Fold change plots illustrating differential mRNA expression of T-BET or EOMES transduced *versus* control transduced HPC. Blue and red dots indicate T-BET- and EOMES-specific differential genes, respectively. Orange dots indicate genes differentially regulated by both overexpression constructs. Selected differential genes are highlighted. **(B)** Bar charts showing the number of differential events of RNA- and ATAC-seq for T-BET- or EOMES-overexpressing *versus* control HPC. **(C)** Overlap analysis of differential ATAC sites in HPC overexpressing T-BET *versus* EOMES. **(D)** NK cell-linked ATAC sites were defined as differential ATAC sites of stage 4 control NK cells *versus* control HPC. These were then compared to the differential ATAC regions of T-BET- or EOMES-overexpressing *versus* control HPC. Bar charts show relative numbers of ATAC sites. **(E)** Overlap analysis of up- or downregulated mRNA and the indicated subgroups of ATAC sites for T-BET- or EOMES-overexpressing HPC. Relative numbers of ATAC sites are shown in the bar chart. **(C–E)** Up = upregulated sites; down = downregulated sites; both = up- and downregulated sites; no diff = detectable sites without differential expression compared to control cultures; none = non-detectable sites. **(F, G)** Motif enrichment analysis of up- or downregulated, or non-differential ATAC sites of T-BET- or EOMES-overexpressing HPC. **(F)** The top 5 best matched motifs are indicated. **(G)** The relative presence of the indicated motif families is shown. **(H)** Representative Genome Browser views of selected gene loci, showing tracks of ATAC-seq for the indicated HPC samples and stage 4 control NK cells, and H3K27ac and/or H3K4me3 ChIP-seq of HPC and mature NK cells. Differentially regulated ATAC regions are indicated in grey or orange, whereby the orange colored regions contain a T-BOX motif. **(I)** Gene set enrichment analysis (GSEA) shows a significant enrichment of CD56^dim^ signature genes in T-BET- or EOMES-overexpressing *vs*. control HPC. Normalized enrichment scores (NES) and q-values are indicated. See also **Supplementary Figure 1**.

Separate overexpression of T-BET or EOMES was obtained by retroviral transduction in human umbilical cord blood (UCB)-derived CD34^+^ HPC, in parallel to the control vector, only containing the eGFP-reporter gene. To evaluate the direct and early effects of T-BET and EOMES overexpression, eGFP^+^lineage^-^CD34^+^ HPC were sorted after 48 h of transduction (hereafter referred to as day 0) and transcriptome and chromatin accessibility profiling were performed by RNA- and Fast-ATAC sequencing, respectively. The gating strategy of the HPC sort is depicted in **Supplementary Figure 1A**. Flow cytometric analysis confirmed the expression of both proteins in the transduced cells (**Supplementary Figure 1B**). mRNA expression analysis revealed that genes expressed in HPC or involved in quiescence of hematopoietic stem cells were downregulated, including *CD34* and *HOPX* (33). Also, genes involved in erythropoiesis, platelet formation or myeloid differentiation, including *TAL1, LMO2, MMRN1* and *MPO* (34–36), were downregulated in T-BET- and/or EOMES-overexpressing HPC. In contrast, a large set of genes encoding transcription factors with a proven role in murine and/or human NK cell development was upregulated, including *ETS-1, IKZF2, IKZF3, ZNF683, IRF8, TOX, TOX2, TXNIP, TCF7* and *ZBTB16* (3, 8, 32, 37–39). In addition, genes encoding NK cell developmental and/or cytotoxicity markers were upregulated as well, including *IL2RB, KLRD1, SLAMF7, ITGAL, NCR3, CD244, PRF1, GZMB, GZMH* and *FASLG* (**Figure 1A**). Differential gene expression of selected genes was confirmed by RT-qPCR or flow cytometry (**Supplementary Figures 1C, D**). The transcriptome profiles of T-BET- and EOMES-overexpressing HPC were partially overlapping, yet subtle differences exist, e.g. *MMRN1, ETS-1, TOX2, PRF1* and *FASLG* were exclusively differentially regulated by EOMES overexpression, while *TCF7* and *ZBTB16* were only upregulated with T-BET overexpression (**Figure 1A**; **Supplementary Table 1**). Overall, more differentially expressed genes were identified in EOMES-overexpressing (727 UP, 698 DOWN) compared to T-BET-overexpressing HPC (333 UP, 239 DOWN) (**Figure 1B**, upper part). The same trend was observed in the number of differential ATAC sites, whereby EOMES-overexpressing HPC had noticeably more differential ATAC sites (18283 UP, 10591 DOWN) compared to T-BET-overexpressing HPC (12817 UP, 5978 DOWN) (**Figure 1B**, lower part). The majority of the T-BET-induced up- and downregulated ATAC regions were similarly regulated upon EOMES overexpression. However, a relatively larger part of EOMES-induced up- and downregulated ATAC regions was not differentially regulated by T-BET overexpression (**Figure 1C**). To investigate whether T-BET or EOMES overexpression in HPC regulates the overall chromatin accessibility of NK cell-linked genes, we first defined the NK cell-linked ATAC regions by comparing control stage 4 NK cells to control HPC, that were then compared to differential ATAC sites of T-BET- or EOMES-overexpressing HPC *versus* control HPC. The results show indeed that the majority of more and less accessible NK cell-linked ATAC regions corresponded to up- and down-regulated ATAC regions, respectively, upon T-BET or EOMES overexpression in HPC (**Figure 1D**).

Analysis of the overlap between the transcriptome and chromatin accessibility profile of HPC showed that the majority of the upregulated genes upon T-BET or EOMES overexpression had either non-differential or upregulated ATAC sites, or contained both up- and downregulated ATAC sites. A minority of these genes contained downregulated ATAC sites only. Whereas a higher fraction of genes with downregulated mRNA expression displayed downregulated ATAC sites, a relatively high fraction also contained non-differential or upregulated ATAC sites (**Figure 1E**). Mainly T-BOX motifs, followed by RUNX and ETS motifs, were present in upregulated ATAC regions upon T-BET or EOMES overexpression, whereas mainly ETS motifs, followed by GATA and RUNX motifs, were prevalent in downregulated ATAC regions (**Figures 1F, G** and **Supplementary Table 2**)

We inspected the chromatin landscape of selected genes that were up- or downregulated in HPC upon T-BET or EOMES overexpression, and combined this analysis with available H3K27ac ChIP-seq data (40, 41). Somewhat unexpected, HPC-linked genes downregulated upon T-BET or EOMES overexpression, like *CD34* and *HOPX*, showed particular DNA regions that became more accessible and these open regions were also present in stage 4 control NK cells. This indicates that these ATAC regions are presumably important for the repression of *CD34* and *HOPX* transcription. Other downregulated genes involved in non-NK lineage differentiation showed less accessible DNA regions in T-BET- or EOMES-overexpressing HPC, whereby these particular DNA regions were closed in stage 4 control NK cells and the majority of these regions also lacked a T-BOX DNA binding motif (**Figure 1H**; upper part). Genes of several NK cell-linked transcription factors and developmental markers showed upregulated ATAC sites in HPC upon T-BET or EOMES overexpression, and these DNA regions were also accessible in stage 4 control NK cells. Comparison to available mature NK cell-derived H3K4me3 and H3K27ac histone-ChIP data (41), that respectively reveal active promotors and poised or active promotors and enhancers, shows that promotors and/or enhancers were activated upon T-BET or EOMES overexpression. The large majority of these promotors and enhancers contained a T-BOX DNA binding motif (**Figure 1H**; middle part), in sharp contrast to the less accessible DNA regions, as mentioned above (**Figure 1H**; upper part). Genes involved in NK cell cytotoxicity also showed putative enhancer and/or promotor regions regulated by T-BET or EOMES overexpression in HPC (**Figure 1H**; lower panel). Gene set enrichment analysis (GSEA) further demonstrated that the transcriptome of T-BET or EOMES overexpressing HPC was highly enriched for mature CD56^dim^ NK cell specific transcripts (**Figure 1I**).

Altogether, these results indicate that T-BET and EOMES overexpression in HPC results in epigenetic changes in the chromatin landscape of NK cell-linked genes and induces an NK-cell specific transcriptome. The effects of T-BET or EOMES overexpression on the transcriptome and chromatin accessibility of HPC are largely overlapping, in which EOMES plays a more pronounced role.

### NK Cell Differentiation Upon T-BET or EOMES Overexpression in Human HPC Is Drastically Accelerated

**Figure 2:**
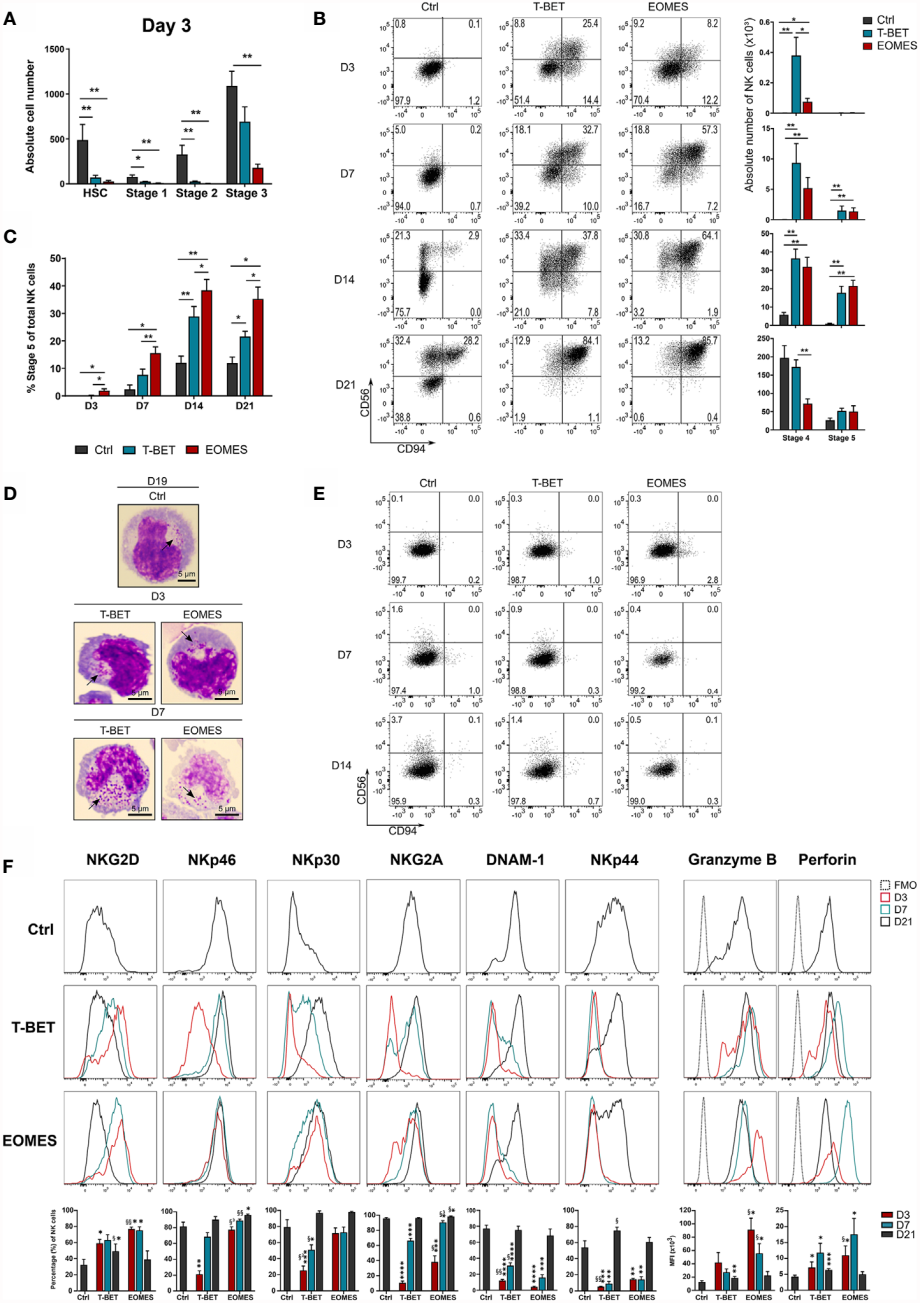
NK cell differentiation upon T-BET or EOMES overexpression in human HPC is drastically accelerated. T-BET, EOMES or control transduced HPC were cultured in the *in vitro* NK cell differentiation co-culture. Cultures were analyzed by flow cytometry on pre-gated eGFP^+^CD45^+^ cells on different time points as indicated. **(A)** Absolute cell numbers of HSC (CD34^+^CD45RA^-^), stage 1 (CD34^+^CD45RA^+^CD117^-^), stage 2 (CD34^+^CD45RA^+^CD117^+^) and stage 3 cells (CD34^-^CD117^+^CD94^-^) at day 3 of culture (mean ± SEM; n = 5-6). **(B)** Representative dot plots of CD11a^+^ gated cells at the indicated days of culture. Cells in the upper right quadrant represent the CD56^+^CD94^+^ NK cell population. The numbers in the plots indicate the percentages. Absolute cell numbers of stage 4 (CD16^-^) and stage 5 (CD16^+^) NK cells are shown in the bar charts (mean ± SEM; n = 6-9). **(C)** Percentages of stage 5 (CD16^+^) of total NK cells is shown (mean ± SEM; n = 6-9). **(D)** Microscopic images of sorted CD56^+^CD94^+^ NK cells on day 3 and 7 of overexpression cultures and on day 19 of control cultures. Arrows indicate cytotoxic granules. 100x magnification. Scale bar = 5µm. **(E)** Representative dot plots of CD11a^+^ gated cells from cultures in the absence of exogenous IL-15. **(F)** Representative histograms of the indicated NK cell markers of gated NK cells (CD11a^+^CD56^+^CD94^+^), whereby the overexpression conditions at different days of culture are compared to day 21 control NK cells. The fluorescence minus one (FMO) is included as background fluorescence. Percentages or MFI of the indicated marker is presented in the bar charts (mean ± SEM; n = 6-12). *, **, *** and **** indicate significant difference of the specified overexpression condition compared to the control, with p<0.05, <0.01, <0.001, <0.0001, respectively. §, §§ and §³ indicate significant difference of T-BET *vs*. EOMES overexpression, with p<0.05, <0.01, 0.001, respectively. See also **Supplementary Figure 2**.

Transduced and eGFP^+^-sorted HPC were cultured in NK cell differentiation conditions for 21 days. Kinetic analysis of T-BET or EOMES expression in NK cells generated from control *versus* T-BET- or EOMES-overexpressing HPC demonstrated that T-BET and EOMES expression was maintained throughout the 21 day culture period (**Supplementary Figure 2A**). After 3 days of culture, nearly no HSC (CD34^+^lineage^-^CD45RA^-^), stage 1 (CD34^+^CD45RA^+^CD117^-^) and stage 2 (CD34^+^CD45RA^+^CD117^+^) cells remained in the T-BET and EOMES overexpression cultures, whereas these populations were still clearly present in control transduced cultures. In addition, significantly less stage 3 (CD34^-^CD117^+^CD94^-^) cells were found upon EOMES overexpression in comparison to the control (**Figure 2A**). In sharp contrast, mature stage 4 (CD56^+^CD94^+^CD16^-^) NK cells already appeared at day 3 of T-BET and EOMES overexpression cultures, whereas these cells merely become detectable at day 14 of control cultures (**Figure 2B**). In addition, maturation towards stage 5 (CD56^+^CD94^+^CD16^+^) NK cells was also accelerated and increased throughout the overexpression cultures, where these cells were clearly present from day 7 compared to few cells at day 14 in control cultures (**Figure 2B** and **Supplementary Figure 2B**). Throughout the culture, the percentage of CD16-expressing cells with T-BET overexpression was significantly increased compared to the control condition, but was highest with EOMES overexpressing cells (**Figure 2C** and **Supplementary Figure 2B**). Microscopic imaging of sorted CD56^+^CD94^+^ NK cells from T-BET or EOMES overexpression cultures at day 3 and 7 of culture, showed the clear presence of cytotoxic granules, resembling an NK cell morphology (**Figure 2D**).

Because T-BET and EOMES overexpression in HPC increased the speed of NK cell development, the possibility existed that T-BET or EOMES overexpression overruled the need for IL-15, a cytokine driving NK cell differentiation, maturation and survival through IL2Rβ signaling (3). However, cultures in the absence of exogenous IL-15 showed no NK cell generation in T-BET or EOMES overexpression cultures (**Figure 2E**).

To further phenotype these early arising NK cells, a more in-depth kinetic analysis of the expression of activating and inhibitory NK receptors was performed on gated CD56^+^CD94^+^ cells from days 3, 7 and 21 overexpression cultures and were compared to day 21 control NK cells (**Supplementary Figure 2C**). The earliest receptor expressed on NK cells generated from T-BET- or EOMES-overexpressing HPC was NKG2D, with even higher NKG2D expression on days 3 and 7 compared to control NK cells. Expression of the activating receptors NKp46 and NKp30, and the inhibitory receptor NKG2A gradually increased to control levels in the T-BET cultures, whereas expression in EOMES cultures was already high from day 3 (NKp46 and NKp30) or day 7 (NKG2A). DNAM-1 and NKp44 expression was delayed and control levels were reached from day 21 in both overexpression conditions. Finally, the cytotoxic effector proteins, perforin and granzyme B, were early expressed in both overexpression cultures, whereby the expression of these proteins was even higher on days 3 and 7 compared to control NK cells (**Figure 2F**).

In conclusion, T-BET and EOMES overexpression in HPC drastically accelerates NK cell generation, whereby these NK cells have a granular morphology, display early or gradual expression of NK receptors and early expression of perforin and granzyme B. In this model, EOMES is a stronger driver of the early NK cell phenotype and a better enhancer of CD16 expression than T-BET. Nevertheless, NK cell differentiation in T-BET or EOMES overexpression conditions remains IL-15 dependent.

### Differentiating NK Cells Upon T-BET or EOMES Overexpression Undergo Terminal NK Cell Maturation, Whereby T-BET Plays a Predominant Role

**Figure 3:**
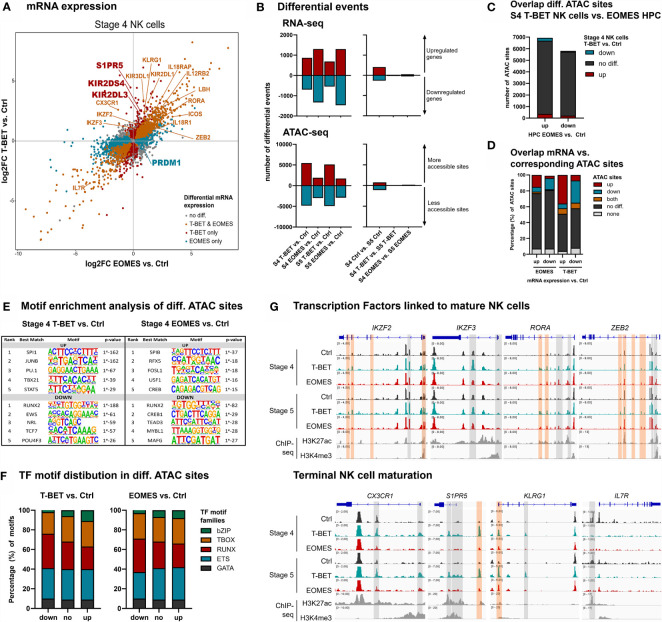
NK cells developing from T-BET- or EOMES-overexpressing HPC undergo terminal NK cell maturation. eGFP^+^CD45^+^CD56^+^CD94^+^ stage 4 (CD16^-^) and stage 5 (CD16^+^) NK cells were sorted from day 21 cultures and RNA-seq (n=4) and Fast-ATAC-seq (n=2) was performed. **(A)** Fold change plots illustrating differential mRNA expression of T-BET or EOMES *versus* control stage 4 NK cells. Blue and red dots indicate T-BET- and EOMES-specific differential genes, respectively. Orange dots indicate genes differentially regulated by both overexpression constructs. Selected differential genes are highlighted. **(B)** Charts show the number of differential events of RNA- and ATAC-seq for T-BET or EOMES stage 4 and stage 5 NK cells compared to control NK cells (left panel) and for stage 4 compared to stage 5 NK cells (right panel). **(C)** Overlap analysis of differential ATAC sites of T-BET *vs.* control stage 4 NK cells and of EOMES-overexpressing *vs*. control HPC. Charts demonstrate the absolute number of ATAC sites. **(D)** Overlap analysis of up- or downregulated mRNA and the indicated subgroups of ATAC sites for T-BET or EOMES *versus* control stage 4 NK cells. Relative numbers of ATAC sites are shown in the bar charts. **(C, D)** Up = upregulated sites; down = downregulated sites; both = up- and downregulated sites; no diff = detectable sites without differential expression; none = non-detectable sites. **(E, F)** Motif enrichment analysis of up- or downregulated, or non-differential ATAC sites of T-BET or EOMES compared to control stage 4 NK cells. **(E)** The top 5 best matched motifs are indicated. **(F)** The relative presence of the indicated motif families is shown. **(G)** Representative Genome Browser views of selected gene loci, showing tracks of ATAC-seq for the indicated stage 4 and stage 5 NK cells, and of H3K27ac and H3K4me3 ChIP-seq of mature NK cells. Differentially regulated ATAC regions are indicated in grey or orange, whereby the orange colored regions contain a T-BOX motif. See also **Supplementary Figures 3** and **4**.

To obtain an in-depth analysis of the effects of T-BET or EOMES overexpression in mature NK cells, RNA and Fast-ATAC sequencing was performed on isolated stage 4 (CD16^-^) and stage 5 (CD16^+^) NK cells from day 21 control, T-BET and EOMES overexpression cultures (**Supplementary Figure 3A**). Transcriptome analysis of stage 4 NK cells upon T-BET or EOMES overexpression showed upregulation of *IKZF2, IKZF3, RORA, PRDM1* and *ZEB2*, that are transcription factor genes known to be involved in terminal NK cell maturation (3, 42–45). In addition, *CX3CR1, S1PR5* and *KLRG1*, characteristic for terminal NK cell maturation (45, 46), were exclusively upregulated in stage 4 and/or stage 5 NK cells from T-BET overexpression cultures (**Figure 3A** and **Supplementary Figure 3B**). Furthermore, *IL7R*, which is known to be down-regulated in CD56^dim^ *versus* CD56^bright^ NK cells (47), was also downregulated in stage 4 NK cells from both T-BET and EOMES overexpression cultures (**Figure 3A**). Comparable transcriptional differences between T-BET- or EOMES-overexpression conditions *versus* control cells were found in stage 5 NK cells (**Supplementary Figure 3B**). Importantly, when comparing the transcriptome of stage 5 *versus* stage 4 NK cells, there were 671 differentially expressed genes in the control cultures, whereas there were only 54 and 102 differentially expressed genes in the T-BET- and EOMES-overexpression cultures, respectively (**Figure 3B**, right panel; **Supplementary Table 3**). These findings suggest that stage 4 NK cells upon T-BET or EOMES overexpression share many similarities with stage 5 control NK cells, further confirming terminal NK cell maturation. Similar to the results in HPC, more genes were differentially expressed in NK cells from EOMES compared to T-BET cultures, in both stage 4 and stage 5 NK cells (2629 *vs.* 1564 and 2763 *vs.* 1238 genes, respectively) (**Figure 3B**, left panel; **Supplementary Table 4**). However, the opposite was true for the number of differential ATAC sites, which was significantly lower in NK cells upon EOMES overexpression compared to T-BET overexpression (4876 *vs.* 10 278 and 4601 *vs.* 10 013 sites in stage 4 and stage 5, respectively) (**Figure 3B**, left panel). As indicated above, EOMES-overexpressing HPC had more differential ATAC sites than T-BET-overexpressing HPC (**Figure 1B**), which raised the possibility that EOMES overexpression already regulates the chromatin accessibility of genes in HPC that only become regulated by T-BET overexpression from stage 4 NK cells. However, as overlap analysis showed that most ATAC sites that are differential in T-BET-overexpressing *vs.* control stage 4 NK cells are not differential in EOMES-overexpressing *vs*. control HPC (**Figure 3C**), this is not the case. This implicates, as expected, that chromatin accessibility continues to be altered during HPC differentiation towards stage 4 NK cells. Analysis of the overlap between the transcriptome and chromatin accessibility profile of stage 4 NK cells showed differences between the T-BET- and EOMES-overexpression cultures. While in cells from T-BET-overexpression cultures a considerable part of the genes with up- or downregulated transcription contained up- or downregulated ATAC sites, respectively, this was considerably less evident upon EOMES overexpression. Here, more than 70% of the ATAC sites was not differentially regulated compared to control NK cells (**Figure 3D**), indicating that transcription is considerably less regulated by epigenetic changes on the chromatin level. Finally, in contrast to the findings in HPC (**Figure 1E**), few differentially expressed genes contained both up- and downregulated ATAC sites (**Figure 3D**). Similar results were obtained with stage 5 NK cells (**Supplementary Figure 3C**). Mainly ETS motifs, followed by T-BOX and RUNX motifs were present in upregulated ATAC regions of stage 4 and stage 5 NK cells generated upon T-BET or EOMES overexpression in HPC, while predominantly RUNX motifs followed by ETS and T-BOX motifs were prevalent in downregulated ATAC sites (**Figures 3E, F** and **Supplementary Figures 3D, E** and **Supplementary Table 5**).

Detailed inspection of the chromatin landscape of *IKZF2, IKZF3, RORA* and *ZEB2*, that encode transcription factors linked to mature NK cell differentiation (3, 42–45) and that showed increased mRNA expression in NK cells upon T-BET or EOMES overexpression, revealed that promotor and/or putative enhancer sites became more accessible (**Figure 3G**; upper panel). In sharp contrast, the accessibility of promotor and/or putative enhancer sites of *CX3CR1*, *S1PR5 and KLRG1* exclusively increased upon overexpression of T-BET (**Figure 3G**; lower panel) and this correlated with the increased mRNA expression in T-BET, but not in EOMES overexpression cultures. Conversely, *IL7R* displayed downregulated ATAC sites, corresponding to decreased mRNA expression (**Figure 3G**; lower panel). Corresponding to the motif enrichment analysis (**Figures 3E, F**), T-BOX DNA binding motifs were less prevalent in the upregulated ATAC regions (**Figure 3G**), in contrast to HPC accessible ATAC regions whereby T-BOX motifs were predominant (**Figure 1H**).

**Figure 4:**
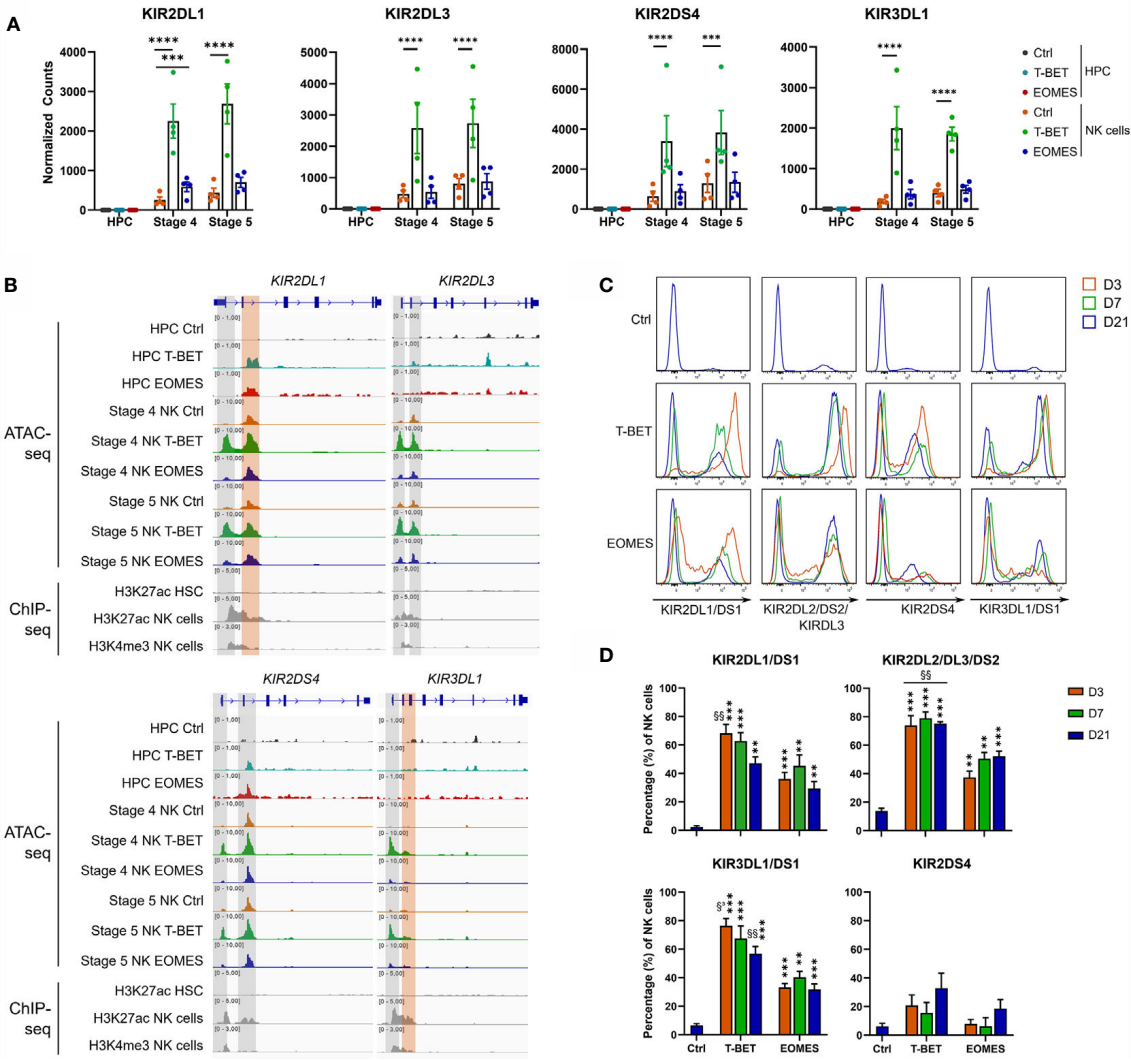
T-BET overexpression epigenetically regulates increased KIR expression in NK cells. **(A)** Normalized counts (mean ± SEM; n = 4-5) are shown from the RNA-seq analysis of the indicated KIR genes in day 0 HPC and day 21 stage 4 and stage 5 NK cell populations. The dots represent individual donors. **(B)** Representative Genome Browser views of the indicated KIR genes, showing tracks of ATAC-seq for all above mentioned cell populations and of H3K27ac and/or H3K4me3 ChIP-seq of HPC and mature NK cells. **(C, D)** Day 21 T-BET, EOMES or control NK cells were analyzed by flow cytometry for KIR expression on different time points as indicated. Representative histograms are shown in **(C)**, percentages of KIR^+^ cells in the NK cell population are demonstrated in **(D)** (mean ± SEM; n = 5-8). "**, *** and **** indicate a significant difference compared to the control with p<0.01, p<0.001 and p<0.0001, respectively. §§ and §³ indicate a significant difference of T-BET vs. EOMES overexpression, with p<0.01 and p<0.001, respectively.

Further analysis of the transcriptome showed that there was no detectable KIR mRNA expression in control HPC, whereas there was a gradual increase in control stage 4 and stage 5 NK cells, as expected. Neither T-BET nor EOMES overexpression induced KIR mRNA in HPC. Whereas EOMES overexpression did not affect (KIR2DS4 and KIR2DL3) or slightly increased (KIR2DL1 and KIR3DL1) KIR mRNA expression in stage 4 and stage 5 NK cells, T-BET overexpression resulted in a drastic increase of all these KIR mRNAs (**Figure 4A**). Strikingly, the chromatin landscape revealed an upregulated ATAC region in KIR2DL1 and KIR2DS4 genes in HPC overexpressing T-BET or EOMES, although no mRNA was expressed. These ATAC regions were also present in control stage 4 and stage 5 NK cells. In stage 4 and stage 5 NK cells upon T-BET or EOMES overexpression, promotor regions of *KIR2DL1, KIR2DL3, KIR2DS4* and *KIR3DL1* were more accessible compared to control NK cells, whereby these ATAC regions were predominantly upregulated by T-BET overexpression (**Figure 4B**). Kinetic analysis by flow cytometry showed that both the expression level and percentage of *KIR2DL1, KIR2DL3, KIR2DS4* and *KIR3DL1* in NK cells was drastically upregulated from day 3 in T-BET overexpression cultures, and to a lesser extent in EOMES overexpression cultures, in comparison to control cultures at day 21 (**Figures 4C, D**). In contrast to NK cells from day 21 control cultures, where as expected stage 4 cells expressed lower KIR levels than stage 5 cells, there was no clear difference in KIR expression in stage 4 *vs* stage 5 NK cells from T-BET or EOMES overexpression cultures (data not shown).

In the *in vitro* NK cell co-cultures, probably due to the activating effect of IL-15, CD56 expression is increased on all NK cells and CD56^bright^ and CD56^dim^ cells cannot be distinguished. We determined the mean fluorescence intensity (MFI) of CD56 in gated NK cells (CD56^+^CD94^+^) at different time points of culture. At day 3 and 7 of culture, NK cells from T-BET or EOMES overexpression cultures had a significantly lower CD56 MFI as compared to NK cells from day 21 control cultures. At day 21, CD56 expression levels decrease from stage 4 to stage 5 for all conditions. The CD56 MFI of NK cells from EOMES overexpression cultures was similar to that of control cultures. In contrast, day 21 T-BET overexpression NK cells showed a significant lower CD56 MFI (**Supplementary Figure 4A**). Also expression of *NCAM1*, coding for CD56, was significantly downregulated upon T-BET overexpression and not upon EOMES overexpression in day 21 NK cells, in both stage 4 and stage 5 NK cells. Moreover, *NCAM1* expression was significantly downregulated when comparing T-BET *vs.* EOMES overexpression in day 21 NK cells (**Supplementary Figure 4B**). We also evaluated other markers of terminally differentiated NK cells. NK cells from all conditions at day 21 lacked CD57 expression, probably due to *in vitro* culture conditions. As expected, KLRG1 expression was lower in stage 4 compared to stage 5 NK cells in all conditions, whereby T-BET overexpression stage 5 NK cells tended to have the highest expression of KLRG1 (data not shown). Altogether, these findings are in agreement with increased terminal differentiation of T-BET overexpressing NK cells.

In conclusion, the transcriptome, chromatin accessibility and protein expression profile gave insights on how T-BET and EOMES epigenetically regulate NK cell maturation and subsequently activate the terminal NK cell transcriptome, wherein T-BET plays a more potent role.

### T-BET and EOMES NK Cells Have Increased Functionality and EOMES NK Cells Display Enhanced ADCC

**Figure 5:**
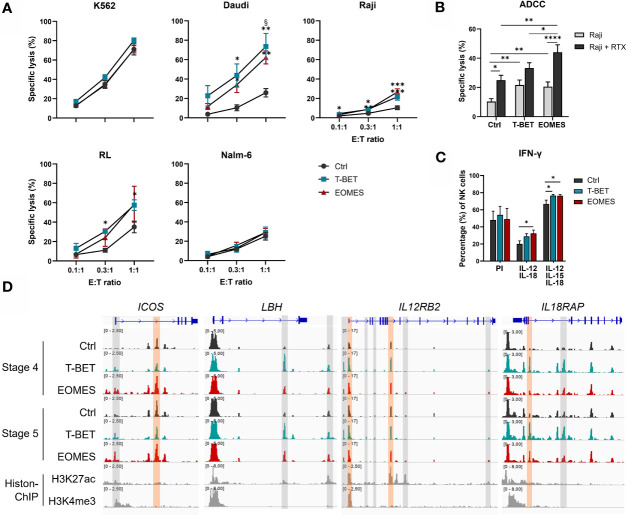
T-BET and EOMES NK cells are functional and EOMES NK cells have enhanced ADCC activity. **(A, B)** Sorted day 21 NK cells (eGFP^+^CD45^+^CD11a^+^CD56^+^CD94^+^) of control, T-BET and EOMES overexpression conditions were challenged with the indicated cell lines at different effector over target (E:T) ratios. **(A)** The percentage of specific target cell lysis is shown (mean ± SEM; n = 2-15). **(B)** ADCC assay with Raji target cells in the absence or presence of Rituximab (RTX) (E:T ratio = 1:1). The percentage specific target cell lysis is shown (mean ± SEM; n=15). **(C)** Intracellular IFN-γ production after 24 h stimulation with PMA and Ionomycin (PI) or with a combination of IL-12 and IL-18 in the presence or absence of IL-15. Bar charts represent the percentage of IFN-γ^+^ cells in the gated NK cell population (mean ± SEM; n=6). **(A–C)** *, **, *** and **** indicate a significant difference, with p<0.05, p<0.01, p<0.001 and p<0.0001, respectively. § indicates a significant difference of T-BET vs. EOMES overexpression, with p<0.05. **(D)** Representative Genome Browser views of the indicate genes, showing tracks of ATAC-seq in stage 4 and stage 5 NK cells from both overexpression and control samples, and H3K27ac and H3K4me3 ChIP-seq of mature NK cells. Colored ATAC regions are differentially regulated and only the regions indicated in orange contain a T-BOX motif. See also **Supplementary Figure 5**.

An important function of human NK cells is cytotoxic killing of malignant or virus-infected cells. NK cells from day 21 overexpression and control cultures were challenged with several target cell lines. K562 cells, the prototypic NK cell susceptible target cell line (48), was similarly killed by NK cells from all conditions. Additionally, the human B cell lymphoma cell lines Daudi, RL and Raji, were only killed to a low extent by NK cells from control cultures, whereas killing by NK cells from T-BET or EOMES overexpression cultures was significantly increased. In contrast, Nalm-6, another human B cell line, displayed resistance to efficient killing by NK cells from all culture conditions (**Figure 5A**).

In addition to direct recognition of tumor targets, NK cells also lyse malignant cells upon binding of tumor cell-bound antibody by the CD16 receptor expressed on stage 5 NK cells, a process known as ADCC (49). Substantially more CD16^+^ NK cells were obtained with NK cells from T-BET and EOMES overexpression cultures (**Figures 2B, C**), whereby both the percentage as well as the level of CD16 expression was higher in EOMES overexpression cultures (**Figure 2B** and **Supplementary Figure 5A**). Therefore, the ADCC capacity was tested using the CD20-expressing human Burkitt’s lymphoma cell line Raji in the absence or presence of Rituximab, a humanized monoclonal anti-CD20 antibody (50). The results show that inclusion of Rituximab increased killing, as expected, but importantly, NK cells from EOMES overexpression cultures displayed significantly higher ADCC functionality as compared to the control or T-BET overexpression conditions (**Figure 5B**). The chromatin landscape of *FCGR3A*, the gene encoding CD16 in NK cells (49), was highly similar in NK cells from all culture conditions (**Supplementary Figure 5B**), indicating that increased CD16 expression upon T-BET or EOMES overexpression is not epigenetically regulated.

Another crucial function of NK cells is production of pro-inflammatory cytokines, including IFN-γ, by which NK cells regulate other immune cells. IFN-γ production upon PMA/Ionomycin stimulation of day 21 NK cells was comparable between conditions. However, IL-12/IL-18 or IL-12/IL-15/IL-18 stimulation resulted in significantly higher IFN-γ production of NK cells upon T-BET or EOMES overexpression compared to control NK cells (**Figure 5C**). The transcriptome profile showed that NK cells from T-BET- or EOMES-overexpression cultures had upregulated mRNA expression of *ICOS, LBH, IL12RB2, IL18R1* and *IL18RAP* (**Figure 3A**). These NK cells also displayed increased accessibility of promotor and enhancer regions in *ICOS* and *IL12RB2*, whereas enhancer regions in *LBH* and *IL18RAP* were upregulated mainly by NK cells from T-BET overexpression cultures (**Figure 5D**).

These findings show that NK cells generated from T-BET- or EOMES-overexpressing HPC have increased cytotoxic capacity against certain tumor targets and NK cells from EOMES overexpression cultures have enhanced ADCC capacity. The NK cells generated upon T-BET or EOMES overexpression display increased IFN-γ production upon cytokine stimulation and this increase is potentially epigenetically regulated.

## Discussion

Overexpression of T-BET or EOMES in UCB-derived HPC alters the chromatin landscape, correlating with loss of an HPC-related transcriptome and induction of an NK cell transcriptome. Subsequent NK cell differentiation is accelerated, with increased ADCC potential in EOMES cultures and increased KIR expression in T-BET cultures.

T-BET and EOMES are the signature transcription factors defining murine and human mature NK cells, but information on their role in human NK cell differentiation is limited. Murine studies have shown that Eomes and T-bet play a crucial role in early and late NK cell differentiation, respectively. Mouse NK cells mature in a 4-stage developmental program, whereby CD27^low^CD11b^low^ cells develop successively *via* CD27^+^CD11b^low^ and CD27^+^CD11b^+^ intermediate stages into CD27^low^CD11b^+^ terminally mature NK cells (51). Eomes- or T-bet-deficient mice display reduced NK cell numbers, wherein Eomes-deficient mice show less differentiation past the CD27^low^CD11b^low^ stage, whereas in T-bet^-/-^ mice NK cell development is halted at the CD27^+^CD11b^+^ stage. T-bet/Eomes double-deficient mice have a complete block in NK cell development, indicating that these highly homologous T-box transcription factors also have non-redundant functions (16, 17). During murine NK cell maturation, the levels of Eomes and T-bet inversely correlate and this is consistent with Eomes acting upstream of T-bet in the NK cell maturation cascade (17, 52). Chromatin accessibility and transcriptome profiling following T-BET or EOMES overexpression in HPC provide novel insights on the regulatory role of these transcription factors in humans. Both T-BET and EOMES downregulated HPC-linked genes and genes involved in erythropoiesis, platelet formation or myeloid differentiation, whereas they upregulated genes of NK cell-linked transcription factors, membrane receptors and effector proteins. Overall, more differentially expressed genes were identified in EOMES-overexpressing compared to T-BET-overexpressing HPC. Interestingly, ETS-1, which has a proven role in both murine and human NK cell development (32, 53, 54), was only upregulated upon EOMES overexpression. The effects of T-BET and, more pronounced, of EOMES on the transcriptome were reflected in the chromatin landscape. It was striking that a large part of the more accessible ATAC regions contained a T-BOX motif, suggesting transcriptional regulation by T-BET or EOMES, whereas in the majority of downregulated ATAC regions ETS, GATA and RUNX motifs were prevalent. In this context, there is ample evidence that ETS, GATA and RUNX transcription factors bind to T-BET and/or EOMES, thereby inhibiting or inducing DNA binding of these co-factors (55–59).

T-BET or EOMES overexpression both accelerated differentiation of HPC into NK cells, but our kinetic NK cell receptor analysis showed that NKp46, NKp30 and NKG2A were readily expressed upon EOMES overexpression, while expression was delayed upon T-BET overexpression. Furthermore, EOMES was a better enhancer of CD16 expression in NK cells, whereby both the percentage of CD16-expressing cells as well as the level of CD16 expression was higher as compared to T-BET overexpression. Altogether, this indicates that also in human predominantly EOMES has a role during development of HPC into CD16-expressing NK cells. This is compatible with studies showing that a T-BET/EOMES gradient is present during human NK cell maturation, wherein EOMES is expressed prior to T-BET. T-BET upregulation during further NK cell differentiation is mirrored by EOMES downregulation, whereby EOMES^low^T-BET^high^ NK cells are terminally mature (18, 19, 60).

In human NK cells, KIR upregulation correlates with decreased EOMES and increased T-BET levels, with highest KIR expression in EOMES^low^T-BET^high^ terminally mature NK cells (14, 19). Our data show that, although NK cells from EOMES overexpression cultures had increased KIR expression compared to control NK cells, KIR expression was highest in T-BET overexpression cultures. This was reflected in increased chromatin accessibility and in higher mRNA and protein levels. The functional homologs of KIRs are Ly49 receptors in mice. Eomes-deficient mice exhibit a limited repertoire of Ly49 receptors, with decreased expression of Ly49A/D/G2/H, whereas T-bet-deficient NK cells express a normal repertoire of activating and inhibitory receptors, with a higher proportion of Ly49D and Ly49G2 (16, 17). Our data show that although EOMES can establish KIR expression in human NK cells, T-BET is superior in this regard. This might implicate that during physiological terminal human NK cell differentiation T-BET further increases KIR expression that is initiated by EOMES. Our data further show that both chromatin accessibility and transcription of genes known to be involved in terminal maturation, including *ZEB2, CX3CR1, S1PR5* and *KLRG1* (43, 45, 46). were upregulated upon T-BET overexpression. As shown in mice and suggested in human (16–19, 52), our data are in agreement with a crucial role of T-BET in terminal NK cell maturation.

Proper NK cell functionality is mandatory for an effective immune response against malignant cells. NK cells generated upon T-BET or EOMES overexpression in HPC showed efficient killing of NK-sensitive K562 cells and increased killing of Daudi, RL and Raji tumor cell lines. The latter cell lines are partially resistant to NK cell-mediated lysis as they express HLA-class I molecules and/or lack ligands for activating NK receptors, like NKG2D (48, 61, 62). Intrinsic potential of discrete NK cell subsets is established during NK cell differentiation and it has been shown that terminally mature subsets of NK cells display superior ability to kill target cells in comparison to less mature subsets (63).

NK cells from EOMES cultures additionally showed enhanced ADCC potential, that correlated with increased CD16 expression. This was a surprise, as *ex vivo* stage 5 NK cells express CD16 and display ADCC functionality and these cells are mainly EOMES^low^T-BET^high^. However, there is more complexity in our culture model as NK cells from EOMES overexpression cultures also express endogenous T-BET, and the reverse is true for NK cells from T-BET overexpression cultures. This is clear from the kinetic analysis of EOMES and T-BET expression, as shown in **Supplementary Figure 2A**. This kinetic analysis also shows that, whereas ectopic EOMES expression is very high from day 0 to day 7, it equals the endogenous EOMES expression of control cells at day 14 and it even becomes lower than endogenous EOMES expression of the control cells at day 21. Our hypothesis is that the high ectopic expression of EOMES early in the culture programs the cells for increased ADCC capacity once the ectopic EOMES expression decreases later on.

CD16 expression is crucial in NK cell activation against tumor cells *via* ADCC. *In vitro* generated NK cells from induced pluripotent or UCB-derived stem cells, commonly used as a source of NK cells for adoptive cell transfer therapy, display low levels of CD16 expression (64–67). Attempts to increase CD16 expression of these NK cell products proves its importance in adoptive NK cell therapy (67). The importance of CD16 is further illustrated by therapeutic approaches focusing on monoclonal antibodies against tumor targets, in which the Fc-receptor regions are modified to increase CD16-binding and subsequently improve ADCC activity of the transferred NK cells (68, 69). The synergistic effects of tumor-targeting monoclonal antibodies and transfusion of NK cells have been shown and are further investigated in ongoing clinical trials (70, 71). Moreover, CD16 expression facilitates not only the combination of transferred NK cells with tumor-targeting monoclonal antibodies to exploit ADCC, but it also plays a crucial role in functionality of Bi- or Tri-specific killer engagers containing a single-chain scFv against the tumor antigen and CD16 (72). Therefore, strategies to improve the ADCC potential of NK cells provide a valuable tool to optimize adoptive NK cell therapies.

NK cells are potent producers of pro-inflammatory cytokines, including IFN-γ (73). The early burst of IFN-γ secretion is not impaired in murine T-bet-deficient NK cells, but after 24 h of stimulation significantly less IFN-γ is produced. This suggests that the presence of T-bet is important for a positive feedback loop on IFN-γ expression (16, 17). A possible role for Eomes in inducing IFN-γ in the absence of T-bet is seen in murine CD8 T cells (74). IFN-γ is a direct target of T-bet in NK cells, whereas no evidence exists for Eomes-binding to the IFN-γ locus (16). In human, CD56^bright^ NK cells efficiently produce IFN-γ after IL-12/IL-18 stimulation, which correlates with increased *IL12RB2* and *IL18RA* mRNA expression (60). Our results show that NK cells generated upon T-BET or EOMES overexpression in HPC produced significantly more IFN-γ after IL-12/IL-15/IL-18 stimulation in comparison to control NK cells. Besides, these NK cells displayed upregulated mRNA expression of *ICOS, LBH, IL12RB2* and *IL18RAP*, with increased accessibility of promotor and/or enhancer regions of these gene loci. During NK cell maturation, epigenetic alterations in signaling components occur (3). T-bet is known to recruit JMJD3 and SET7 in Th1 cells which generate chromatin remodeling of the *IL12RB2* gene, leading to gene expression (55). Also the *IL18R1/IL18RAP* gene locus is a T-bet target in T cells (75). The role of EOMES in regulating signaling components during NK cell activation is less clear. The transcriptional activator LBH activates activator protein-1 (AP-1) and is thus involved in the MAPK-pathway employed in IL-18 signaling (76), although a role for LBH in NK cells is currently unknown. A recent study revealed that ICOS plays and important role in NK cell functionality, whereby ICOS-deficient mice display lower IFN-γ levels after *in vivo* stimulation (77). Both transcriptional regulators possibly attribute to the increased IFN-γ production in our T-BET or EOMES cultures, due to enhanced downstream signaling after cytokine stimulation.

Altogether, our findings give molecular insights in the regulatory role of T-BET and EOMES upon overexpression in human HPC and subsequent NK cell differentiation, and can be used to optimize adoptive NK cell therapies.

## Data Availability Statement

The datasets presented in the study are accessible on GEO with accession number GSE166439 or can be found on https://www.ncbi.nlm.nih.gov/geo/query/acc.cgi?acc=GSE166439.

## Ethics Statement

The usage of human umbilical cord blood in this study was reviewed and approved by the Ethics Committee of the Faculty of Medicine and Health Sciences, Ghent University, Ghent, Belgium. The patients/participants provided their written informed consent to participate in this study.

## Author Contributions

Conceptualization: LK and GL. Methodology: LK, ST, SW, EP, EA, and GL. Investigation: LK, ST, SW, EP, EA, and ZV. Formal Analysis: LK. Software: WL. Visualization: LK and WL. Supervision: GL. Writing – original draft: LK and GL. Writing – review and editing: LK, ST, SW, EP, ZV, PM, FN, TT, PV, BV, and GL. All authors contributed to the article and approved the submitted version.

## Funding

The computational resources (Stevin Supercomputer Infrastructure) and services used in this work were supplied by the VSC (Flemish Supercomputer Center), funded by Ghent University, FWO and the Flemish Government – department EWI. This work was supported by grants from the Research Foundation - Flanders (FWO) [G.0444.17N (GL), 1S29317N (LK), 12N4515N (ST) and 1S45317N (SW)] and Kinderkankerfonds (a non-profit childhood cancer foundation under Belgian law to PV and WL).

## Conflict of Interest

An international patent application WO2020070070 was filed by Ghent University (Ghent, Belgium) on 30/09/2019, with title ‘Accelerated human hematopoietic stem cell differentiation towards mature natural killer cells with enhanced antibody-dependent cytotoxic activity’, with LK and GL as inventors.

The remaining authors declare that the research was conducted in the absence of any commercial or financial relationships that could be construed as a potential conflict of interest.

## Publisher’s Note

All claims expressed in this article are solely those of the authors and do not necessarily represent those of their affiliated organizations, or those of the publisher, the editors and the reviewers. Any product that may be evaluated in this article, or claim that may be made by its manufacturer, is not guaranteed or endorsed by the publisher.
