# Supplementary material for: T-BET and EOMES Accelerate and Enhance Functional Differentiation of Human Natural Killer Cells
Source: Front Immunol. 2021 Sep 24;12:732511. doi: 10.3389/fimmu.2021.732511 (PMC8497824; doi:10.3389/fimmu.2021.732511)
Supplement: Supplementary file 1 [file DataSheet_1.pdf]

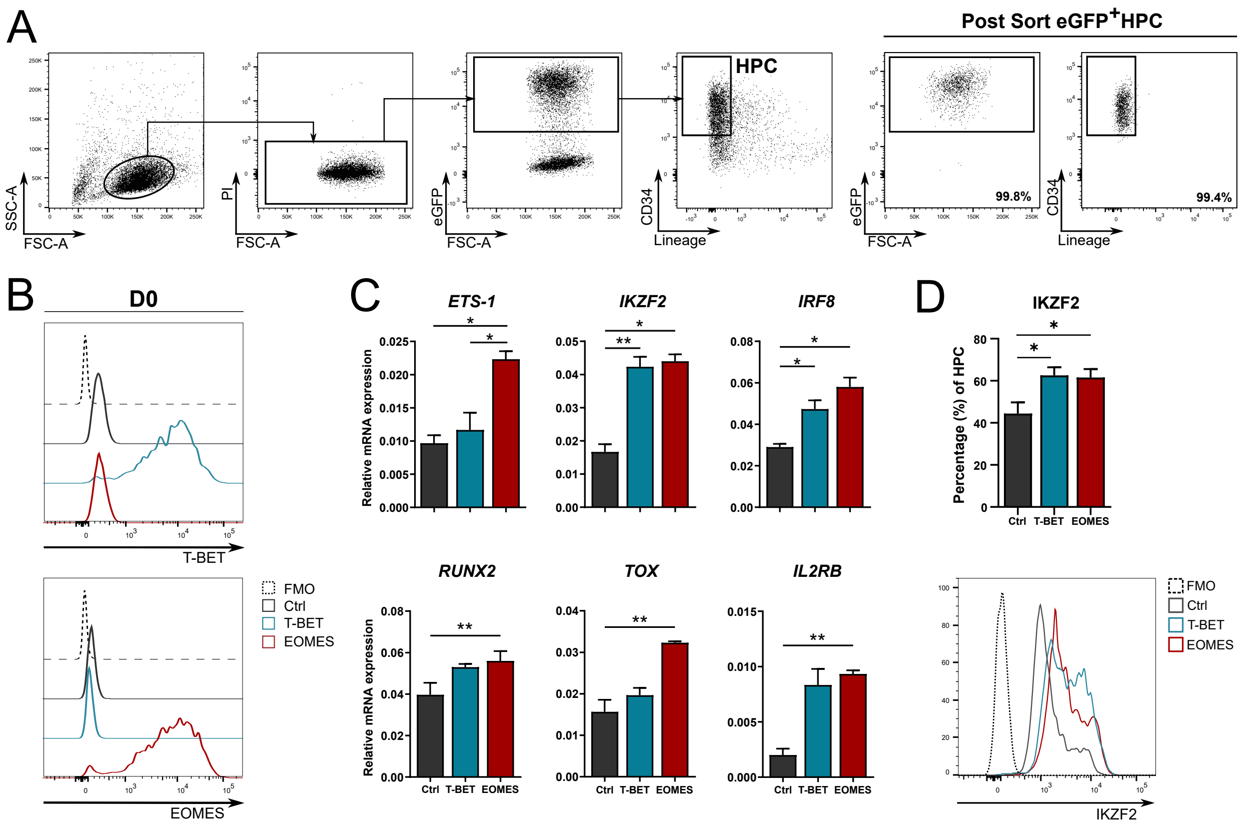

Supplementary Figure 1. Confirmation of T -BET and EOMES protein expression and transcriptome profiling in HPC upon T-BET or EOMES overexpression.

(A) Representative dot plots showing the gating strategy to sort HPC (eGFP^{+}Lineage^{-}CD34^{+}) for RNA- and ATAC -seq. Purity after sort is indicated in the post sort plots as percentages. PI = propidium iodide. Lineage = combined staining of CD3, CD14, CD19 and CD56**.** **(B)** T-BET and EOMES protein expression was analysed by flow cytometry 48 h after transduction (day 0) of both overexpression and control conditions. The dotted line shows the fluorescence minus one (FMO). **(C)** RT-qPCR analysis of selected genes that were differentially expressed in the RNA-seq analysis of T-BET- or EOMES-overexpressing versus control HPC. The relative mRNA expression is shown (mean ± SEM; n=3). * and ** indicate a significant difference with p<0.05 and p<0.01, respectively **(D)** Analysis of IKZF2 protein expression in the indicated HPC populations by flow cytometry. The upper panel demonstrates the percentage of IKZF2^{+} HPC (mean ± SEM; n=3). * indicates significant difference (p<0.05). Representative histograms are show in the lower panel.

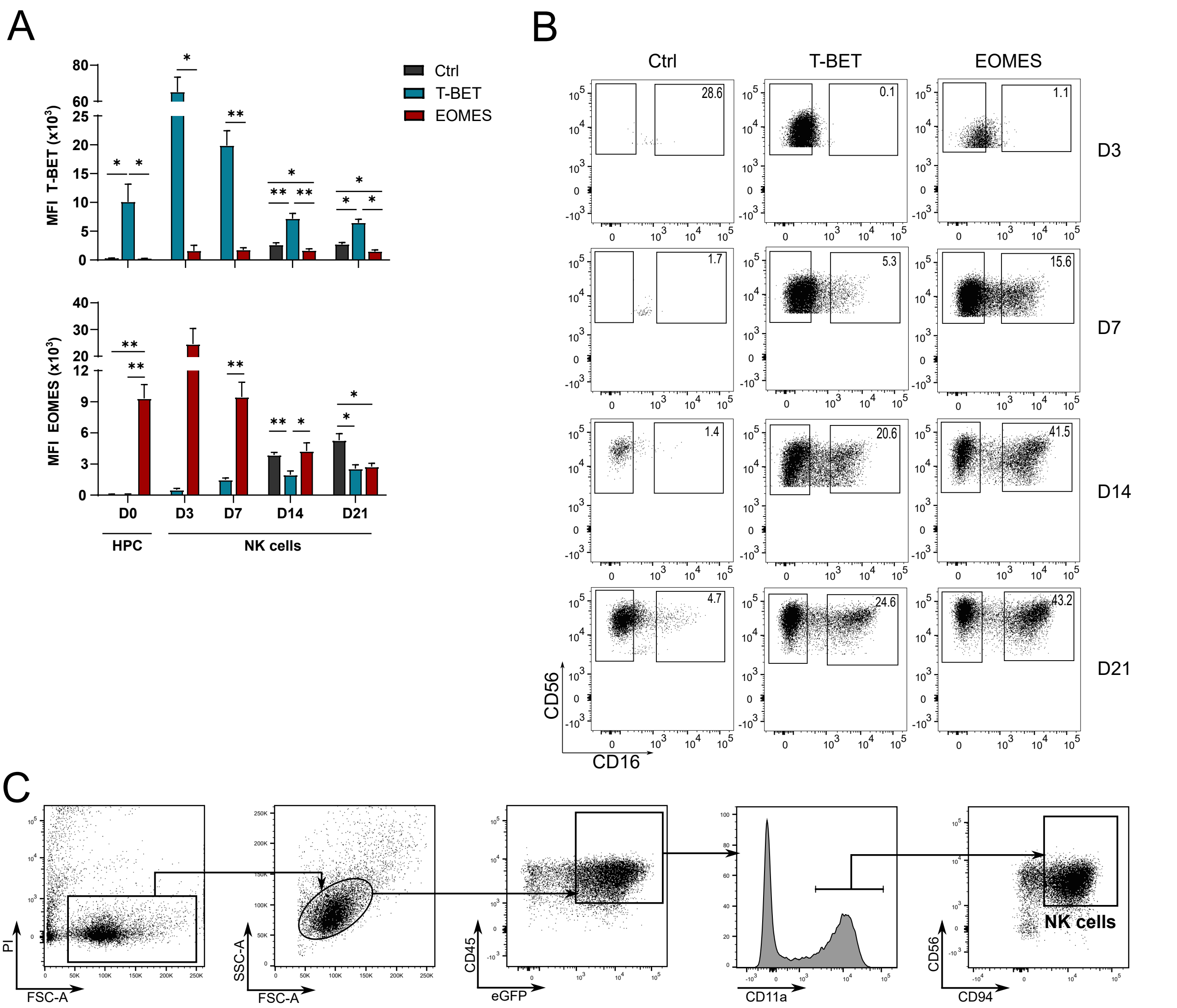

Supplementary Figure 2. Kinetic analysis of T-BET and EOMES expression in generated NK cells and stage 5 NK cell generation upon T-BET or EOMES overexpression in HPC. **(A)** T-BET and EOMES protein expression levels were analysed by flow cytometry in gated HPC or NK cells at the indicated time points,and shown as mean fluorescence intensity (MFI) (mean ± SEM; n = 4-10). * and ** indicates statistical significance with $p<0.05$ and $p<0.01$, respectively. **(B)** Representative dot plots of gated (eGFP$^+$CD45$^+$CD11a$^+$CD56$^+$CD94$^+$) NK cells analysed on the indicated time points. Cells in the left and right gate are stage 4 (CD16$^-$) and stage 5 (CD16$^+$) NK cells,respectively. Numbers in the right gate indicate the percentages of stage 5 NK cells. **(C)** Representative dot plots showing the gating strategy for mature NK cell (eGFP$^+$CD45$^+$CD11a$^+$CD56$^+$CD94$^+$) to evaluate the NK cell receptor repertoire. PI=propidium iodide.

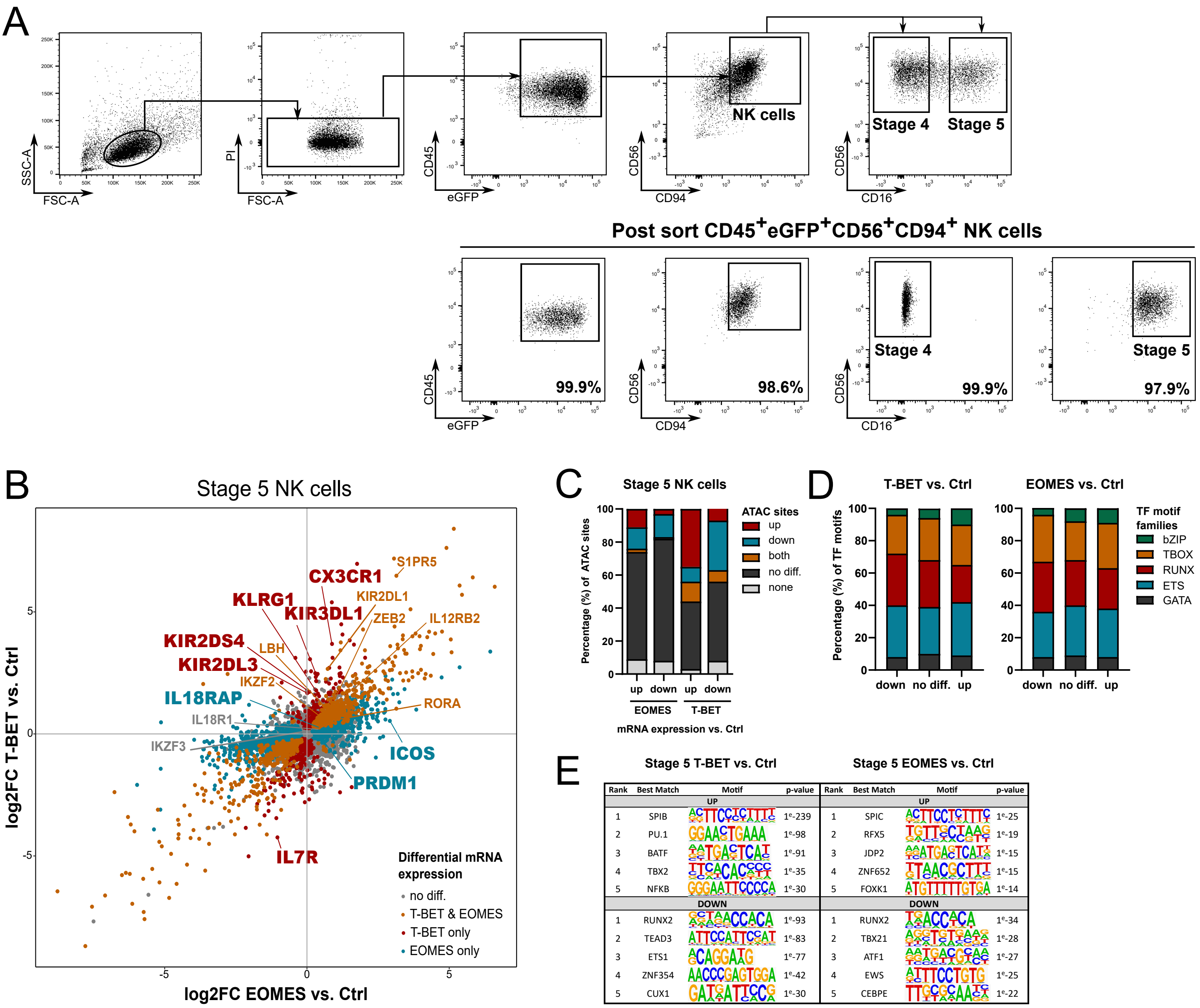

Supplementary Figure 3. Transcriptome and chromatin accessibility profiling of stage 5 NK cells upon T-BET and EOMES overexpression. (A) Representative dot plots showing the gating strategy to sort mature stage 4 (eGFP$^+$CD45$^+$CD56$^+$CD94$^+$ CD16$^-$) and stage 5 (eGFP$^+$CD45$^+$CD56$^+$CD94$^+$ CD16$^+$) NK cells for RNA- and ATAC-seq. Post sort analysis is depicted in the lower panel with the percentages of purity of each population indicated in the plots. PI = propidium iodide. **(B-D)** Stage 5 (eGFP$^+$CD45$^+$CD56$^+$CD94$^+$ CD16$^+$) NK cells were sorted from day 21 cultures and RNA-seq (n=4) and Fast-ATAC seq (n=2) was performed. **(B)** Fold change plots demonstrating differential mRNA expression of T-BET or EOMES versus control stage 5 NK cells. Red and blue dots indicate T-BET- and EOMES-specific differential genes, respectively. Orange dots indicate genes differentially regulated by both overexpression conditions. Selected differential genes are highlighted. **(C)** Overlap analysis of up- or downregulated mRNA and the indicated subgroups of ATAC sites for T-BET or EOMES versus control stage 5 NK cells. Relative numbers of differential ATAC sites are shown in the bar charts. Up = upregulated sites ; down = downregulated sites; both = up-and downregulated sites; no diff= detectable sites without differential expression; none = non-detectable sites. **(D-E)** Motif enrichment analysis of up- or downregulated, or non-differential ATAC sites of T-BET or EOMES compared to control stage 5 NK cells. The relative presence of the indicated transcription factor motif families is shown in **(D)** The top 5 best matched motifs are indicated in **(E)**.

A

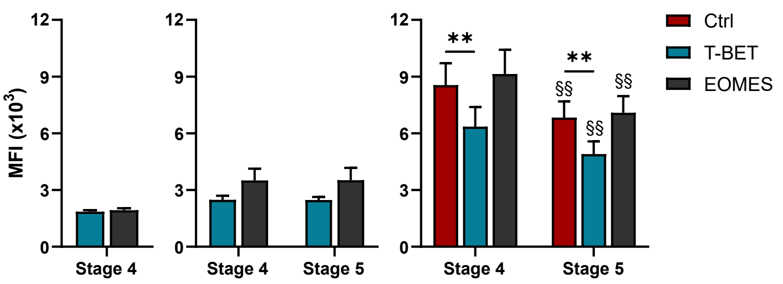

B

NCAM1

	Stage 4		Stage 5	
	Fold change	p-value	Fold change	p-value
T-BET vs. Ctrl	-0,399	0,000286	-0,429	9,52E-05
EOMES vs. Ctrl	0,125	0,392426	0,122	0,390048
T-BET vs. EOMES	-0,52	1,10E-09	-0,55	1,17E-08

Supplementary Figure 4. Analysis of the CD56 expression levels in NK cells upon T-BET and EOMES overexpression. (A) T-BET, EOMES or control transduced HPC were cultured in the *in vitro* NK cell differentiation co-culture. Cultures were analysed by flow cytometry on pre-gated eGFP$^+$CD45$^+$CD11a$^+$ cells on different time points as indicated. CD56 expression levels for stage 4 (CD56$^+$CD94$^+$CD16$^-$) and stage 5 (CD56$^+$CD94$^+$CD16$^+$) NK cells are displayed as mean fluorescence intentsity (MFI) in the bar charts. ** indicates a significant difference with $p<0.01$. §§ indicates a significant difference with $p<0.01$ of stage 4 vs. stage 5 NK cells. **(B)** Stage 4 (eGFP$^+$CD45$^+$CD56$^+$CD94$^+$CD16$^-$) and stage 5 (eGFP$^+$CD45$^+$CD56$^+$CD94$^+$CD16$^+$) NK cells were sorted from day 21 cultures and RNA-seq(n=4) was performed. *NCAM1* transcript expression, coding for CD56, is shown in the table for the different conditions indicated. P-value < 0.05 is considered significant.

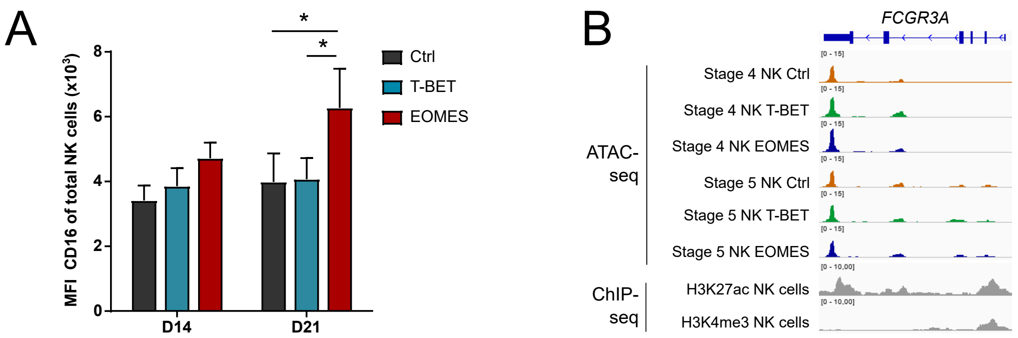

Supplementary Fig. 5. CD16 protein expression levels and chromatin accessibility landscape. (A) Mean fluorescence intentisty (MFI) of CD16 on gated NK cells from day 14 and 21 of culture (mean ± SEM; n = 6-9). * indicates statistical significance ($p < 0.05$). **(B)** Representative Genome Browser views of *FCGR3A*, showing tracks of ATAC -seq in stage 4 and stage 5 NK cells from both overexpression and control samples, and H3K27ac and H3K4me3 ChIP-seq of mature NK cells.
